# Department of Surgery

**Published:** 1901-01

**Authors:** W. E. A. Wyman, J. A. Sloan, Robert Jay

**Affiliations:** Burlington, Wis.; San Francisco, Cal.; West Liberty, Ia.


### DEPARTMENT OF SURGERY.

By W. E. A. Wyman, M.D.V., VS.,
BURLINGTON, WIS.

J. A. Sloan, B.Sc., M.D.V.,
SAN FRANCISCO, CAL.

Robert Jay, M.D.V.,

WEST LIBERTY, IA.

---

### Hoof Diseases.

(Continued from page 760.)

### *Bruises of the Pododerm—Corns.*

Any defined hemorrhage of the pododerm of the quarters, bars, or sole depending on bruising or undue stretching of the pododerm of the above-mentioned region is known under the beautiful misnomer "corn." Referring to the above definition, corns of the wall, bars, or sole are encountered, which, according to the time they have existed, are divided into recent and old corns.

Corns are common in the internal aspect of the fore-hoof, and are caused by any force straining or bruising the pododerm. This is readily seen. The anterior extremities sustain most of the weight, and are primarily exposed to concussions, bruises, strains, assisted again by excessive physiological movements of the hoof, especially marked at the inside quarter, which together with excess of weight usually born by the internal quarter serve to explain the fact that this locality is predisposed to corns. Thus faulty positions and insufficiency of hoof-structure demand consideration as predisposing factors in corns. Corns are often met with in limbs which are base-wide or base-narrow, those which toe-out or toe-in, in the coon-footed or stumpy hoof. In this position one part or another of the hoof is exposed to excessive concussions, and, therefore, bruising and tugging of the pododerm. Consequently, in the base-wide position the inner quarter and in the toe-narrow type the outer portions are likely to be affected.

Dry and brittle hoofs, contracted, crooked, weak-heeled, low, and too high-heeled hoofs, and, finally, flat and pumiced feet, are predisposed to corns; and since the cause of the hemorrhage—that is, on the one hand, the weakness of the hoof, and, on the other, the unyielding nature—is difficult to correct, chronic or incurable corns are met here.

Of external causes the preparation of the hoof by the farrier previous to shoeing is of importance. Unless the hoof is properly balanced laceration of the pododerm is only a question of time. The essentials are a correct proportion between length of quarters and toe—in other words, a level-bearing surface. Often the heels are cut too low or one quarter is lower than its mate; the latter is often done with the intention to relieve pressure, while in reality the opposite effect follows this practice, and as the low quarter no longer is braced, the weight of the body crowds it in a downward direction, and besides other sequelæ productive of pain, and thus lameness, the pododerm is stretched, pulled at, and lacerated. Excessive paring of frog and sole weakens those parts until they no longer can execute their physiological duties.

As a direct cause of corns the shoe itself is next of interest. Either by direct pressure or by hindering the physiological movements of the hoof, lacerations and bruises of the pododerm are created; of course, the shoe with calks is primarily concerned in the production of corns, since by it the solar surface of the hoof is unable to support its share of weight bearing, as frog and sole at the moment of weight bearing descend to excess, thus pulling on the pododerm. Shoes which are too short, too narrow, or too wide, or too little concaved in flat feet give rise to a bruised pododerm. In the rural districts corns are quite common even in good feet, as the shoe often remains on all winter and part of the spring, thereby bruising the sole by its four to six months' permanency. The care of the hoof is in this country but little understood by the laity, as they keep them either too dry or too moist, the former neglect being more readily concerned in corns than the latter, as the unyielding nature of the horn, for instance, in contracted feet is a detrimental factor.

According to Lungwitz, on examination of light bruises the part shows a hyperæmic state of the stratum phylloides and possibly stratum vasculosum. The fine vessels of the laminæ are packed with blood cells, and some swelling and increased redness is observed. The horn production of the rete Malphigii is increased, but shows a light yellow color and fatty, translucent appearance, as it is saturated with a serous exudate and imperfectly keratinized. Rupture of bloodvessels in the stratum phylloides and vasculosum, with separation of the pododerm and the youngest layer of the horn cells, is seen in more serious lacerations of the pododerm.

The little cavity thus created fills with the blood which either enters the horn tubules or by capillary attraction saturates the intertubular horn, to appear in time as red or bluish spots at the solar surface of the hoof as the horn grows down. The sequelæ—as a serous or hemorrhagic pododermatitis, or, in case of infection, suppurative or gangrenous pododermatitis—will be taken up subsequently.

So far the lesions can be permanently corrected unless the causes of it persist, when old or chronic corns arise, which, on account of organic changes in the pododerm or horny capsule, may be beyond reach.

Chronic wall or bar corns, so often seen in contracted or curved-in quarters, exhibit a displaced or bent state of the horny and fleshy laminæ. The secondary laminæ become thinner and longer and switch off the curved primary laminæ almost at a right angle. The blood eventually separates the pododerm from the horny wall, and on such a spot the laminæ have no secondary laminæ, or they are short and thick, while the primary ones are small, others strong and long, running in various directions. The cavity thus created is in time filled with a more or less whitish-yellow, soft, horny mass, produced by the surface of the fleshy laminæ. A peculiar rigid appearance of the horny box in old, chronic corns manifests itself.

In chronic sole corns the microscopical changes are closely related to those just enumerated; the horn tubules are more or less curved, the papillæ more often hypertrophy, more rarely do they shorten. (Eberlein.)

The symptoms of corns, as a rule, are confined to the pin-head to dime-sized dark red, bluish spots in the angle of the bars or along the bearing surface (white line), without lameness, barring chronic corns and the presence of an aseptic or infectious pododermatitis, which exhibit distinct clinical symptoms, as heat on palpation, excessive pulsations of the digital or metacarpal arteries, together with pain expressed by lameness. Thus in chronic corns affecting both forelegs a stilty, groggy gait is observed.

From a forensic point of view it is of interest that the depth of the discoloration of the horn is, as a rule, no criterion for the length of time the diseased state has existed, as a shorter or longer space of time is required for the stained cells to grow down ; only in serious lacerations and in chronic corns the discoloration is traced to the pododerm.

Quite different are the clinical symptoms of chronic corns. First, the groggy, stilty gait, the dislocated heels, and the almost diagnostic ridges which, ring-like, span the hoof well developed at the quarters, where they incline more or less toward the bearing surface of the hoof, and, finally, the contracted or curved-under walls at the quarters, either the result of or cause of these corns, while the sole horn over the diseased parts is usually dry and crumbles.

Serous and hemorrhagic pododermatitis and recent corns may be confounded. Therefore, in the differential diagnosis of these states it is to be remembered that recent corns are not accompanied with clinical inflammatory signs (absence of lameness, no excessive arterial pulsations, little or no pain on palpation or percussion of the hoof), the discolored spots being circumscribed, while in serous or hemorrhagic pododermatitis inflammatory symptoms are present.

In the differential diagnosis of chronic corns, contracted feet and ossification of the lateral cartilages play an important role, the clinical features of which have been fully discussed in my work on *Clinical Diagnosis of Lameness in the Horse.*

The prognosis to be given depends on the causes and pathological lesions existing at the time of examination.

In those animals predisposed to corns by malpositions of the legs or deficient hoofs, as previously spoken of, treatment is difficult, as the cause must persist, and an unfavorable prognosis becomes a necessity. When due to faulty shoeing, improper and excessive work on pavement, rough, frozen ground, etc., or neglected care of the hoof, a favorable prognosis may be given, provided these external causes can be modified and no lesions of intense inflammatory character are present. In chronic corns, where contraction of the hoof is usually present, an unfavorable prognosis is mostly called for.

Prophylaxis is the main therapeutic agent. In the treatment of corns careful shoeing is the leading feature ; it must endeavor to balance the foot and not interfere with its physiological functions. Develop the frog as much as possible. Employ, whenever possible, a smooth shoe—that is, do away with calks. Rubber pads may also be employed to advantage where expense is of little consequence. To prevent excessive pressure, and thus bruises and lacerations of the pododerm in a weak heel, rasp the heel portion of the bearing surface down from one-thirty-second to one-sixteenth of an inch before applying the shoe. Contracted and curved-under quarters are best shod with a bar shoe. Here rubber pads find their greatest field of usefulness. A run to grass also is a good curative means. To cut out corns, to remove their roots, etc., is nonsense—that is, when the cut extends into the pododerm, as in this way and only too often an infectious pododermatitis is artificially produced. Occasional moist dressings to the feet, especially in dry weather, to be followed immediately by an oleaginous hoof dressing to prevent the rapid evaporation of the water just introduced by the swab, linseed poultices, soaking tub, etc., are beneficial, as they prevent the hoof from getting too dry and brittle.

Finally, that neurectomy, under the convenient diagnosis of navicular hoof disease, is employed almost daily to remove lameness from chronic corns, a great many may hesitate to admit, but it nevertheless remains a fact.

